# Effect of the amino chain length and the transformation into citric acid salts of aryl-diphenyl-butenes and ferrocenyl-diphenyl-butenes bearing two dimethylaminoalkyl chains on their antimicrobial activities

**DOI:** 10.1186/2193-1801-2-508

**Published:** 2013-10-04

**Authors:** Karim Jellali, Pascal Pigeon, Fatma Trigui, Siden Top, Sami Aifa, Gérard Jaouen, Mehdi El Arbi

**Affiliations:** Centre de Biotechnologie de Sfax (Université de Sfax), Route de Sidi Mansour Km 6, BP 1177, 3018 Sfax, Tunisia; Chimie ParisTech (Ecole Nationale Supérieure de Chimie de Paris), Laboratoire Charles Friedel, UMR CNRS 7223, 11 rue Pierre et Marie Curie, 75231 Paris Cedex 05, France

**Keywords:** Diaryl butene, Antimicrobial activity, Citrate formulation, Citrate salts resistance, Hydrochlorides formulation

## Abstract

In a previous work we have demonstrated the antimicrobial activity of ferrocenyl or phenyl derivatives of diphenyl butene series. This finding has opened a new area of applications of organometallic compounds.

In order to improve these activities, we have synthesized new organic and organometallic diaryl butene compounds with different lengths of their amino chains. These new compounds, and also their ammonium salts, were tested against man pathogenic microorganisms *Escherichia coli* (ATCC 10536), *Pseudomonas aeruginosa* (ATCC 15442), *Staphylococcus aureus* (ATCC 6538) and *Enterococcus hirae* (ATCC 10541).

It emerged from the tests that the Gram+ bacteria are more sensitive to the compounds than Gram-, and the compounds with 3 carbon amino chains have a better antimicrobial activity than the one having a chain of 2 or 4 carbons.

The transformation of compounds to citrate salts was accompanied by a significant regression of antibacterial activity against *Pseudomonas aeruginosa*, for both organic and ferrocenic molecules. This resistance problem has been solved using hydrochlorides salts rather than citrates one.

## Introduction

According to global risk report published in 2013, antibiotic-resistant infections kill every year 100,000 Americans, 80,000 Chinese and 25,000 Europeans (Spellberg et al. 2011; Howell 2013). In fact, infections cost 21 to 34 Bn US$/year in USA and 1,5 Bn Euro/year in Europe.

The frequency of antibiotic resistance is profoundly different from one European country to another. Europe was subject of an antibiotherapy study (ICAAC), made by the team of Prof Voss, presented in 1992 to the U.S. Congress and published few years later (Voss et al. 1994). This study showed considerable differences in resistance rates of *Staphylococcus aureus* to meticillin (MRSA) between European countries, with rates ranging from 0% in some northern countries (Finland, Denmark, Sweden) to 35% and 40% in some countries that are more "Latin" (Italy, France). In some countries, MRSA studies, (Voss et al. 1994; Goldstein and Acar 1995) show that almost all strains were resistant to other antibiotics, especially quinolones and rifampicin. Resistance of *Staphylococcus aureus* to meticillin increased also in the most medically-advanced countries, even in United States of America (Gaynes et al. 1993). Every year MRSA kills 19,000 USA patients (more than emphysema, HIV/AIDS, Parkinson’s disease, and homicide combined).

Recently a report titled « Global Risks 2013 », published by World economic forum, demonstrates a decrease on the rate of MRSA infections from 2008 to 2011 in the UK, Germany, Belgium, France and Spain to reach a rate included between 10% and 25%, this rate increased in Hungary and Slovakia (25% to < 50%). In Portugal and Romania MRSA infections statistics are the highest with a rate up to 50%. In addition, the increase of resistance rates for Gram negative bacilli such as *Klebsiella pneumoniae* in all Europe has been reported. This germ shows resistance to three classes of antibiotics combination (3rd generation cephalo-sporins, fluoroquinolones and aminoglycosides) with rates included between 1% and up to 50%. A resistance that can be explained by production of β-lactamase with extended spectrum (Sirot et al. 1993; Burwin et al. 1994). Some countries are also faced with epidemic phenomena associated with the two species of gram-negative willingly multiresistant bacilli *Acinetobacter baumannii* and *Stenotrophomonas maltophilia* (Lortholary et al. 1995).

In Spain and France, an increase of pneumococcal resistance to penicillin has been reported (Pallares et al. 1995). This increase is due to the appearance of resistant gram positive cocci like *Pneumococci* (Pallares et al. 1995) and *Enterococci* (Spellberg et al. 2011; Howell 2013).

Clinical resistance is a complex phenomenon and its manifestation is dependent on many factors, but hospitals are not the only places where bacterial resistance can be developed. The use of biocides in agriculture and antibiotics in animal husbandry are two causes that accentuate the problem.

Allergy is one of the unpredictable problems associated with antibiotics use. This hypersensitivity is a drug reaction that is usually related to either the dose or the pharmacological action of the drug (Solensky 2006; Volcheck 2004). It accounts for approximately 6-10% of all adverse drug reactions and the classes of antibiotics that are most commonly associated with allergic reactions are penicillins, cephalosporins, sulfonamides and macrolides (Gruchalla 2003).

To overcome drug resistance and to find new types of drugs, researchers have been exploring the possibility of using transition metal complexes as drugs against diverse diseases (Jaouen 2006; Hillard and Jaouen 2011; Patra et al. 2012; Gasser and Metzler-Nolte 2012; Chavin and Biot 2010; Hartinguer et al. 2009; Omelas 2011).

Ferrocenyl or phenyl derivatives of diphenyl butene series, tamoxifen analogues, known for their antitumor activity (Jaouen et al. 2000), showed also an excellent antimicrobial activity against *Escherichia coli*, *Pseudomonas aeruginosa*, *Staphylococcus aureus* and *Enterococcus hirae* (El Arbi et al. 2011). This finding, supported by published results of an American team regarding the efficacy of tamoxifen against *Candida albicans* (Dolan et al. 2009), opens a new area of applications to this type of compounds.

Bacterial resistance problems, the inefficiency of antibiotics discovered many years ago, the dual role of antibiotics in combating infectious and cancer diseases and the problem of allergy are good reasons to expend more efforts on the discovery of new active molecules.

On the same vision, this work has as objectives the synthesis of various diphenyl butene compounds with two amino chains, the exploration of the most effective structure microbiologically and the best appropriate formulation to make it water-soluble.

## Materials and methods

### Synthesis and characterization of compounds

The synthesis of all compounds was performed under an argon atmosphere, using standard Schlenk techniques. Anhydrous THF (tetrahydrofurane) was obtained by distillation from sodium/benzophenone. Thin layer chromatography was performed on silica gel 60 GF254. Infrared spectra were obtained on FT/IR-4100 JASCO spectrometers (http://www.jascofrance.fr). ^1^H and ^13^C NMR spectra were recorded on a 300 MHz Bruker spectrometer (http://www.bruker.com). Mass spectrometry was performed with a Nermag R 10-10C spectrometer. Elemental analyses were performed by the microanalysis service of CNRS at Gif – sur – Yvette (https://www.imagif.cnrs.fr). The preparative HPLC (high performance liquid chromatography) separations were performed on a Shimadzu apparatus (http://www.shimadzu.fr) with a Nucleodur C18 column (length of 25 cm, diameter of 2.5 cm, and particle size of 10 μm).

### 1-[Bis(4-hydroxyphenyl)methylidenyl]indan 10

Titanium chloride (15.177 g, 8.79 mL, 80 mmoles) was added dropwise to a suspension of zinc powder (7.844 g, 120 mmoles) in dry THF (200 mL) at 0-20°C. The mixture was heated at reflux for 2 hours. A second solution was prepared by dissolving 1-indanone (2.643 g, 20 mmoles) and 4,4'-dihydroxybenzophenone (4.284 g, 20 mmoles) in dry THF (50 mL). This latter solution was added dropwise and the reflux was continued overnight. After cooling, the mixture was poured in water and dichloromethane was added. The mixture was acidified with diluted hydrochloric acid until dark color disappeared and was decanted. The aqueous layer was extracted with dichloromethane and the combination of organic layers was dried over magnesium sulphate. After concentration under reduced pressure, the crude product was chromatographed on silica gel column with a mixture of cyclohexane and ethyle acetate 50/50 as the eluent to give **10** as a white solid with a 79% yield. The characteristics of the product were identical to that of the literature (Kim and Katzenellenbogen 2000).

### 1-[Bis(4-{3-dimethylaminopropoxy}phenyl)methylidenyl]indan 4

Compound **10** (1.886 g, 6mmol), potassium carbonate(8.29 g, 60 mmol) and cesium carbonate (3.91 g, 12 mmol) were stirred in 120 mL of acetone, then 3-dimethylamino-1-propyl chloride hydrochloride (5.69 g, 36 mmol) was added. The mixture was refluxed overnight, cooled and concentrated under reduced pressure. The residue was extracted with a mixture of dichloromethane and water, then was decanted. The organic layer was washed twice with a diluted aqueous solution of sodium hydroxide followed with water, dried over magnesium sulphate then was concentrated under reduced pressure. The mixture was purified by HPLC using a 10% trietylamine solution in methanol to give **4** as oil with a 67% yield.

^1^H NMR (CDCl_3_) : δ 1.87-2.06 (m, 4 H, CH_2_), 2.27 (s, 6 H, NMe_2_), 2.28 (s, 6 H, NMe_2_), 2.41-2.55 (m, 4 H, CH_2_N), 2.93 (s, 4 H, H_indane_), 3.95-4.09 (m, 4 H, CH_2_O), 6.50 (d, J = 7.7 Hz, 1 H, H_arom_), 6.78-6.92 (m, 5 H, H_arom_), 7.01-7.25 (m, 6 H, H_arom_). ^13^C NMR (CDCl_3_) : δ 27.9 (2 CH_2_), 31.6 (CH_2 indane_), 34.8 (CH_2 indane_), 45.8 (2 NMe_2_), 56.7 (CH_2_N), 56.8 (CH_2_N), 66.5 (2 CH_2_O), 114.1 (2 CH C_6_H_4_), 114.9 (2 CH C_6_H_4_), 125.2 (CH_indane_), 125.3 (CH_indane_), 125.9 (CH_indane_), 127.3 (CH_indane_), 130.7 (2 CH C_6_H_4_), 131.4 (2 CH C_6_H_4_), 134.6 (C), 135.6 (C), 136.7 (C), 139.6 (C), 142.0 (C), 147.9 (C), 157.9 (C), 158.4 (C). IR (KBr, ν cm^-1^): 3063, 3033, 2940, 2854, 2813, 2763 (CH_2_, CH_3_). MS (EI, 70 eV) m/z : 484 [M]^+.^, 439, 86 [CH_2_CH_2_CH_2_NMe_2_]^+^, 58 [CH_2_NMe_2_]^+^.

### 1,1-bis-[4-(3-Dimethylamoniumpropoxy)phenyl]-2-ferrocenyl-but-1-ene dichloride 9

Diamino compound **5** (2.2 g, 3.7 mmol) was dissolved into 200 mL of diethyl ether. A 2 M solution of hydrochloric acid in diethyl ether (3.7 mL, 7.4 mmol) was added dropwise into the solution. An orange precipitate was immediately formed. After stirring for 20 min, the mixture was filtered under argon and the obtained orange solid was washed with 3 × 5 mL of diethyl ether and was dried under vacuum giving compound **9** in 59% yield. Crystals contain traces of diethyl ether.

^1^H NMR (DMSO-d_6_) : δ 1.02 (t, J = 7.4 Hz, 3 H, CH_3_), 2.04-2.27 (m, 4 H, 2 CH_2_), 2.44-2.63 (m, 2 H, CH_2_), 2.79 (s, 12 H, NMe_2_H^+^), 3.10-3.30 (m, 4 H, CH_2_N), 3.85 (s, 2 H, C_5_H_4_), 3.97-4.25 (m, 11 H, C_5_H_4_ + C_5_H_5_ + CH_2_O), 6.86 (d, J = 8.0 Hz, 2 H, C_6_H_4_), 6.90-7.01 (m, 4 H, C_6_H_4_), 7.14 (d, J = 8.0 Hz, 2 H, C_6_H_4_). ^13^C NMR (DMSO-d_6_) : δ 16.3 (CH_3_), 24.8 (2 CH_2_), 28.1 (CH_2_), 42.9 (2 NMe_2_H+), 54.9 (2 CH_2_N), 65.6 (CH_2_O), 65.8 (CH_2_O), 68.8 (2 CH C_5_H_4_), 69.6 (2 CH C_5_H_4_), 69.9 (5 CH C_5_H_5_), 86.8 (C C_5_H_4_), 115.1 (2 x 2 CH C_6_H_4_), 130.8 (2 CH C_6_H_4_), 131.3 (2 CH C_6_H_4_), 137.1 (C), 137.4 (C), 137.9 (C), 138.2 (C), 157.5 (2 C) IR (KBr, ν cm^-1^): 3425 (NH), 3029, 2960, 2685, 2510, 2470 (CH_2_, CH_3_).

### Microbial strains

Known and newly synthesized compounds were tested against *Escherichia coli* (ATCC 10536), *Pseudomonas aeruginosa* (ATCC 15442), *Staphylococcus aureus* (ATCC 6538) and *Enterococcus hirae* (ATCC 10541).

All strains were cultured in liquid LB (1% Bactotryptone, 0.5% Yeast extract, 0.5% NaCl).

### Estimation of the antimicrobial effect

The estimation of the antimicrobial effect against microbial strains was performed by the method of micro-dilution in ELISA plates. A 2.54 10^-3^ M stock solutions of the tested products were prepared in DMSO or water, depending on their solubility. In Elisa plates and for each product a series of eight wells containing 100 μl of culture medium with decreasing concentration of the product were prepared by the successive ½ dilution.

A 100 μl of overnight shaking microbial culture, incubated at adequate temperature, depending on bacterial strains, were used to inoculate the plate wells containing different concentrations of compounds. The final concentration of each product, for a series of eight wells was 2.54 10^-4^ M, 1.27 10^-4^ M, 6.35 10^-5^ M, 3.18 10^-5^ M, 1.59 10^-5^ M, 7.94 10^-6^ M, 3.97 10^-6^ M and 1.98 10^-6^ M. The plates were incubated with shaking overnight at the same temperature, depending on bacterial strains, and their OD was measured at 620 nm.

A negative control (uninoculated wells), a positive control (seeded and without antimicrobial compound wells) were prepared under the same experimental conditions.

The inhibitory activity of the tested compounds was calculated according to the formula:

where (x) is the microbial culture containing the inhibitor and (i) is the microbial culture without inhibitor.

## Results

**Figure 1:**
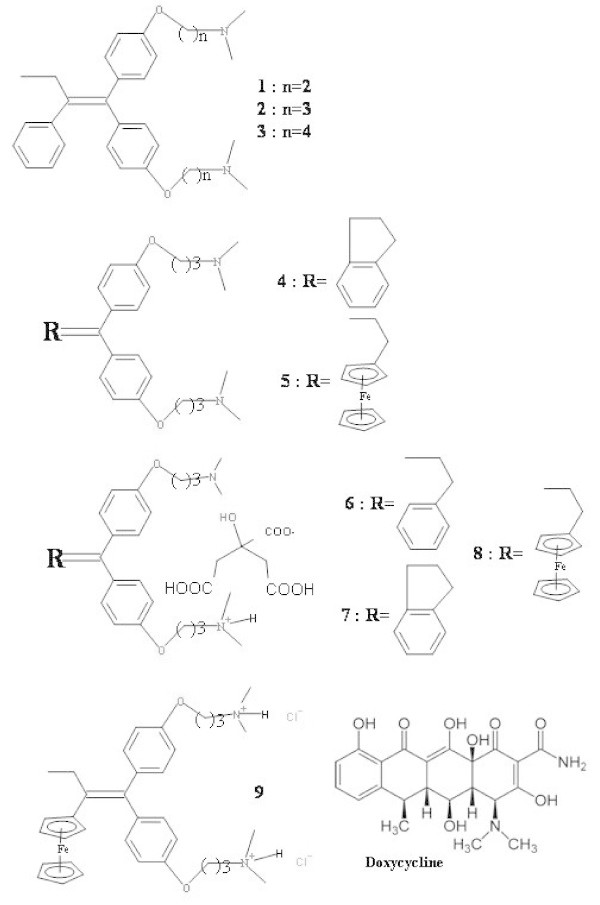
**Chemicals tested.**

All compounds studied in the present work are shown in Figure 1.

### Synthesis

The synthesis of compounds **1** (Shiina et al. 2008), **2** (Pigeon et al. 2011), **5** (Pigeon et al. 2011), and **8** (El Arbi et al. 2011), has already been published. Compounds **3**, **6** and **7** are newly-synthesised products and their synthesis will be published in the future.

**Figure 2:**
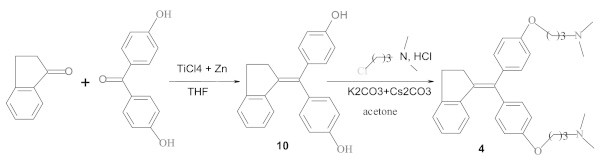
**Synthesis of compounds 4 and 10.**

Figure 2 shows the synthetic pathways of **4**. **10** was first prepared by reacting 1-indanone with 4,4'-dihydroxybenzophenone in the McMurry coupling conditions. This compound was converted into the diamine compound **4** by heating at reflux 3-dimethylamino-1-propyl chloride hydrochloride with **10** in acetone in the presence of potassium carbonate and cesium carbonate as bases. **4** was obtained in the yield of 67%.

**Figure 3:**
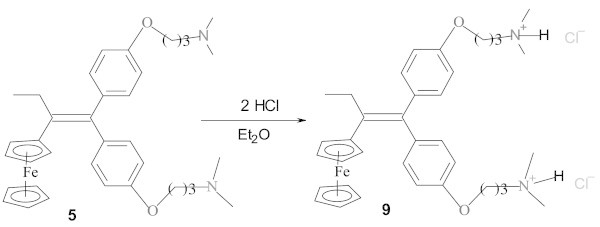
**Synthesis of compound 9.**

Hydrochloride **9** was prepared by adding 2 equivalents of hydrochloric acid to diamine compound **5** dissolved in diethyl ether (Figure 3). **9** was obtained in the yield of 59%.

### Effect of amino chain length on antimicrobial activity

**Figure 4:**
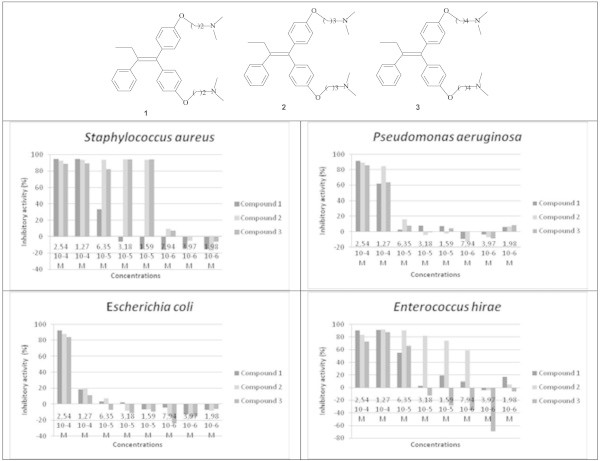
**Antimicrobial activity of compounds 1, 2 and 3.**

To evaluate the impact of the amino chain length on the antibacterial activity, organic compounds **1**, **2** and **3**, having an amino chain of 2, 3 and 4 carbons, respectively, were selected for the test with four bacteria. At a concentration of 2.54 10^-4^M, as shown on Figure 4, all tested strains were sensitive to the three compounds. This result confirms again the activity of compound **3** (El Arbi et al. 2011). The tested compounds are effective against Gram+ and Gram- strains, but not with the same efficiency. Gram+ bacteria, *Staphylococcus aureus* and *Enterococcus hirae*, seem to be more sensitive than Gram- strains, *Escherichia coli* and *Pseudomonas aeruginosa*. Therefore, there is a potential use against Gram+ bacterial infections for these compounds. Within these compounds, the ones with 3 and 4 carbons are more potent comparing to the one of 2 carbons. It is interesting to note that these molecules increase the proliferation of micro-organisms when used at low doses.

### Effect of organic or organometallic compound on the antimicrobial activity

**Figure 5:**
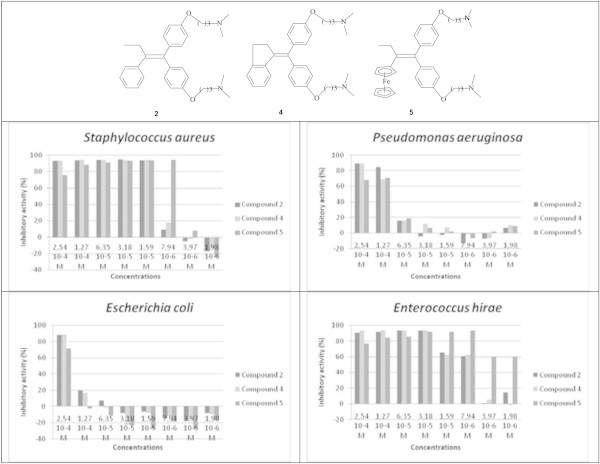
**Antimicrobial activity of compounds 2, 4 and 5.**

It has been shown that ferrocenyl compounds are more active than their organic analogues against cancer cells (Top et al. 2003). It is interesting to see if this behavior is also verified for the antibacterial activity. Therefore, we compare now the activity of two organic compounds, the compounds **2** and **4**, to that of ferrocenic compound **5**. As shown in Figure 5, all compounds are more active against Gram+ than Gram- bacteria. The difference in activity between organic and ferrocenic compounds is clearly observed in the case of *Staphylococcus aureus.* The inhibition percentage (IP) of **2** and **4** is about 94.32% at a dose of 3.59 10^-5^M while that of **5** is 7.94 10^-6^M. In the case of *Enterococcus hirae*, the IP value of 59.21% is obtained using 7.94 10^-6^M for both organic compounds and 1,98 10^-6^M for organometallic compound. Therefore, the organometallic compound **5** is more potent, against Gram+ strains, than the organic compounds (4.5 times for *Staphylococcus aureus* and 4 times for *Enterococcus hirae*).

### Effect of the formulation of salts of citric acid on the antimicrobial activity

**Figure 6:**
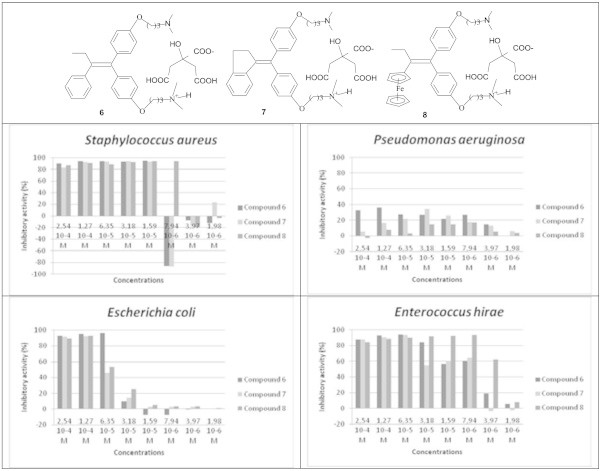
**Antimicrobial activity of compounds 6, 7 and 8.**

Compounds **2**, **4**, **5** are not soluble in water. Their use as antimicrobial agents may be more convenient if they could be transformed into aqueous soluble forms. This transformation was achieved by their conversion into salts of citric acid. Thus, compounds **2**, **4**, and **5** were converted into compounds **6**, **7**, and **8**, respectively. Figure 6 shows that the transformation of the compounds into salts did not alter their activity except for *Pseudomonas aeruginosa*. The activities against *Staphylococcus aureus* and Enterococcus *hirae* were maintained. We even observed an improvement of the antimicrobial activity effect against *Escherichia coli*, compound **6** expressed an IP of 96.2% at a dose of 6.35 10^-5^M while **2** reaches this level of inhibition at a dose of 2.54 10^-4^M. By contrast, the loss of effectiveness against *Pseudomonas aeruginosa* was observed for all salts. Indeed, at the highest dose tested (2.54 10^-4^M), the inhibitory activity decreased from 89%, 89.46% and 63.38% for **2**, **4** and **5**, to 32.7%, 5.6% and -2.7% for **6**, **7**, and **8**, respectively.

### Effect of the transformation to hydrochloride on the antimicrobial activity

**Figure 7:**
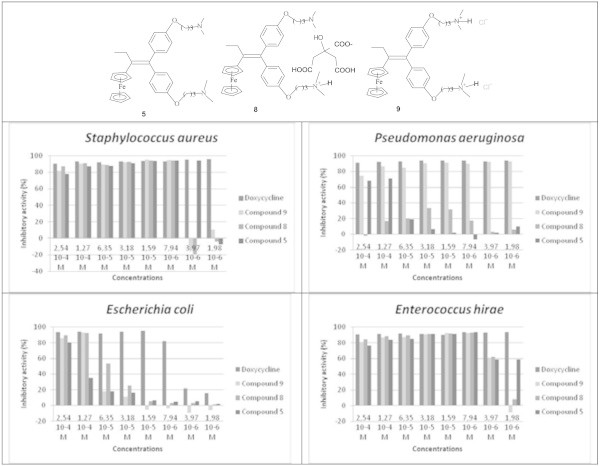
**Antimicrobial activity of compounds 5, 8, 9 and Doxycycline.**

The loss of activity of the salts against *Pseudomonas aeruginosa* prompted us to look for other salts forms of compounds. Thus, compound **5** was converted into hydrochloric salt **9**. Figure 7 shows that **9** preserved both solubility in water and the compound **5** activity observed against *Staphylococcus aureus*, *Enterococcus hirae* and *Escherichia coli*. Interestingly, the loss of activity observed against *Pseudomonas aeruginosa* following the conversion of compound **5** into **8** was recovered by the compound **9**.

Comparing to Doxycycline, an antibiotic of reference, **9** shows a similar activity and has an excellent antibacterial activity, especially against Gram+ (Figure 7).

## Discussion

The present study confirms the importance of using tamoxifen and tamoxifen analogues in microbial inhibition. We found that tamoxifen and ferrocifen derivatives bearing two amino chains are active against bacteria. The activity of compounds can be improved by varying the length of the amino chain, the replacement of one of the arene rings of tamoxifen by a ferrocenyl unit, and by transforming the compounds into aqueous soluble forms. Thus, compounds with 3 carbon amino chain are more active than compounds with 2 and 4 carbon chains. Ferrocene derivatives, the ferrocifen series, have better activity than pure organic compounds (the tamoxifen series). Moreover, transforming the compounds into ammonium salts make the compounds soluble in water and improve the activity of compounds. For this last purpose, hydrochloric salts should be used rather than the citrate ones because of the resistance of *Pseudomonas aeruginosa* of this last salt form.

In addition to its antitumor efficacy, tamoxifen possession of antimicrobial properties has been recently reported (Dolan et al. 2009); meanwhile, knowing that tamoxifen is marketed as tamoxifen citrate, we want to warn following the results the possible ineffectiveness against *Pseudomonas aeruginosa* and the risk of aggravation of nosocomial infections, caused among others, *by Pseudomonas aeruginosa*.

We conclude that it is imperative to avoid the citrate salt formulation for antimicrobial products, because of the loss of compound activity against *Pseudomonas aeruginosa*. We also recommend trying other types of formulation for anti-tumor compounds, especially in the case of chemotherapy treatment, where citrate is used in low doses, which can offer an advantage of proliferative effect for microorganisms and biofilm formation (Robert et al. 2006).
